# Supplementary material for: The correlation between cell and nucleus size is explained by an eukaryotic cell growth model
Source: PLoS Comput Biol. 2022 Feb 18;18(2):e1009400. doi: 10.1371/journal.pcbi.1009400 (PMC8893647; doi:10.1371/journal.pcbi.1009400)
Supplement: S1 Text — (PDF) [file pcbi.1009400.s001.pdf]

Supporting Information: The Correlation Between Cell and Nucleus Size is Explained by an Eukaryotic Cell Growth Model

Yufei Wu[1,5], Adrian F. Pegoraro[2], David A. Weitz[3], Paul Janmey[4], Sean X. Sun[1,5,6,*],

1 Department of Mechanical Engineering, Johns Hopkins University, Baltimore, Maryland
2 Department of Physics, University of Ottawa, Ottawa, Canada
3 Department of Physics, Harvard University, Boston, Massachusetts
4 Department of Cell and Developmental Biology, University of Pennsylvania School of Medicine, Philadelphia, Pennsylvania
5 Institute for NanoBioTechnology, Johns Hopkins University, Baltimore, Maryland
6 Center for Cell Dynamics, Johns Hopkins School of Medicine, Baltimore, Maryland

* ssun@jhu.edu

Comparison between re-scaled parameters and literature-based parameters for mammalian cells

For mammalian cells, most of the parameters are literature-based (except for cytoplasm-nucleoplasm transport coefficients). We also obtained a set of parameters for mammalian cells that are directly scaled up from yeast parameters. The assumption here is that: synthesis and transport rates are proportional to the cell volume and inversely proportional to the cell cycle time. Under this assumption, we can recalculate the parameters for mammalian cells. The results are provided in S1 Table A.

All parameters in rescaled values are close to the literature-based values. Only ribosome synthesis coefficient (s_3) and amino acid transport coefficient (t_1) are slightly different from rescaled values. The similarity and difference between the two sets of parameters can be explained as follows: The protein concentration of yeast cells is the same as that of mammalian cells, therefore scaling up the protein-related parameters from yeast gives the same results as the literature-based results. However, the ribosome concentration and amino acid concentration is higher in yeast than mammalian cells, and this explains why the rescaled values are slightly larger than the literature-based values.

Table A. Model parameters and their numerical estimates.

Parameter	Description	Literature-based Values	Rescaled
n_1	Average number of AA in non-ribosomal proteins	400	400
n_2	Average number of AA in ribosomal proteins	400	400
n_3	Average number of protein subunits in mature ribosomes	80	80
t_1	Amino acid import rate coefficient (h^{-1})	36	45
t_2	Ribosomal protein transport coefficient across nuclear envelop ($\mu\mathrm{m}^3/10^6\mathrm{h}$)	1	1
t_3	Ribosome transport coefficient across nuclear envelop ($\mu\mathrm{m}^3/10^6\mathrm{h}$)	0.15	0.15
t_4	Non-ribosomal protein transport coefficient into nucleus ($\mu\mathrm{m}^3/10^6\mathrm{h}$)	0.1	0.1
t_5	Non-ribosomal protein transport coefficient out of nucleus ($\mu\mathrm{m}^3/10^6\mathrm{h}$)	0.05	0.05
d_{1C}, d_{1N}	Non-ribosomal protein degradation coefficient ($\mu\mathrm{m}^3/10^6\mathrm{h}$)	0.01	0.01
d_{2C}, d_{2N}	Ribosomal protein degradation coefficient ($\mu\mathrm{m}^3/10^6\mathrm{h}$)	0.01	0.01
d_{3C}, d_{3N}	Ribosome disassembly coefficient ($\mu\mathrm{m}^3/10^6\mathrm{h}$)	0.01	0.01
s_1	Non-ribosomal protein synthesis coefficient ($\mu\mathrm{m}^3/10^6\mathrm{h}$)	115	115
s_2	Ribosomal protein synthesis coefficient ($\mu\mathrm{m}^3/10^6\mathrm{h}$)	95	95
s_3	Ribosome assembly coefficient (h^{-1})	1.5	2.4

Analytical approximation of the growth rate and C/N ratio

To obtain analytical results, we further make the following simplifications: (1) assume amino acid is extremely abundant and do not consider the amino acid dynamics. The protein synthesis rate is only proportional to the ribosome number. (2) Nuclear ribosome R_N is much less than cytoplasmic ribosome number R_C (We denote the total number of ribosome to be R so $R_C = R$, $R_N \sim 0$). (3) Transport rate of ribosomal protein is constant (t_2). (4) We only consider the net import of non-ribosomal protein and the rate is set to be proportional to cytoplasmic non-ribosomal protein P_C. Under these assumptions, we obtain:

$$
\begin{aligned}
\frac{dP_C}{dt} &= s_1 R - t_4 P_C, && (1)\\
\frac{dR}{dt} &= \frac{s_3}{n_3} RP_N, && (2)\\
\frac{dRP_C}{dt} &= s_2 R_C - t_2, && (3)\\
\frac{dRP_N}{dt} &= t_2 - s_3 RP_N, && (4)\\
\frac{dP_N}{dt} &= t_4 P_C && (5)
\end{aligned}
$$

These equations can be directly solved as:

$$
\begin{aligned}
P_C &= C_4 e^{-t_4 t} + s_1 \left[\frac{C_1}{n_3(t_4 - s_3)} e^{-s_3 t} + \frac{C_2}{t_4} + \frac{t_2}{n_3 t_4}(t - \frac{1}{t_4}) \right], && (6)\\
R &= \frac{C_1}{n_3} e^{-s_3 t} + \frac{t_2}{n_3} t + C_2, && (7)\\
RP_C &= -\frac{C_1 s_2}{n_3 s_3} e^{-s_3 t} + \frac{t_2 s_2}{2 n_3} t^2 + (C_2 s_2 - t_2) t + C_3, && (8)\\
RP_N &= -C_1 e^{-s_3 t} + \frac{t_2}{s_3}, && (9)\\
P_N &= -C_4 e^{-t_4 t} - \frac{s_1 C_1 t_4}{n_3(t_4 - s_3) s_3} e^{-s_2 t} + s_1 \left(C_2 - \frac{t_2}{n_3 t_4} \right) t + \frac{s_1 t_2}{2 n_3} t^2 + C_5 && (10)
\end{aligned}
$$

Where C_1 C_5 are unknown constants determined by initial conditions. The result here shows that the growth rate is proportional to t^2, which does not capture the feature of exponential growth. We also obtain the steady state $V_C/V_N = 1 + s_2/s_1$ under the assumption that volume coefficients are the same for nucleus and cytoplasm ($r_1 = r_2$). This means the steady state V_C/V_N decreases with s_1 and increases with s_2, which agrees with our numerical results for yeast in Fig 4J. However, this result does not reflect the influence of ribosome synthesis and transport on the C/N ratio, which can be more important.

Linear growth

In the poor nutrient case, we consider growth with protein degradation and ribosome disassembly. In this case, the growth trajectory is not exactly linear, but can be well approximated as linear in the first hundred cell cycles after the 10th cell cycle ($\sim$ 20 h). To illustrate this point, we performed a long-term cell growth simulation in 2000 h without division (which gives the same growth rate as division case), as is shown in the figure below (S1 Fig I):

Mathematically, the solution is not absolutely linear due to degradation. There will be a steady state volume after around 5×10^5 h. However, this is far from the realistic time range we

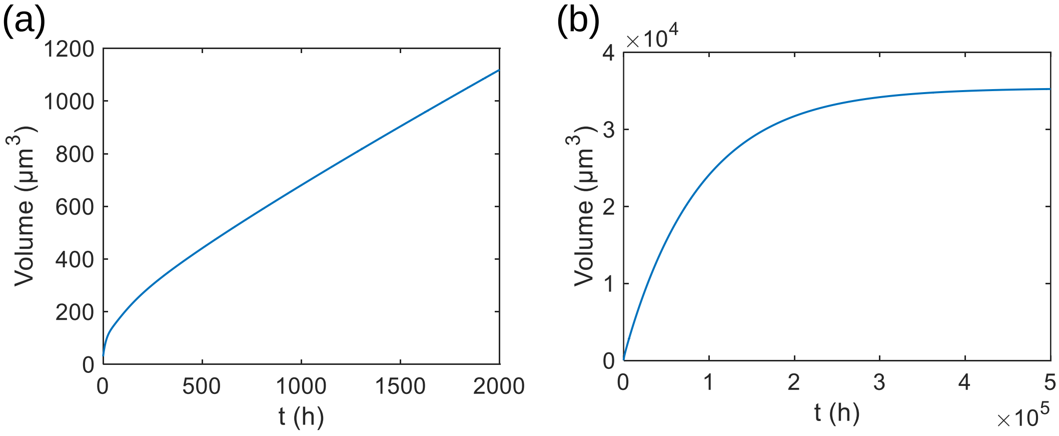

Figure I. Linear growth in the poor nutrient case for very long times.

are considering. Therefore, in realistic times, we can still say that poor-nutrient growth can be well described as linear growth.

Stability of C/N ratio and growth rate

We can test the stability of C/N ratio numerically with different initial conditions and parameters (coefficients of transport, synthesis and degradation). S1 Fig II shows the C/N ratio in yeast (a) and mammalian (b) cells with varying initial conditions (random ±20% of main text values). The numerical result shows that the C/N ratio is generally stable with respect to proteome composition (S1 Fig II). We also test the stability of yeast cell C/N ratio with

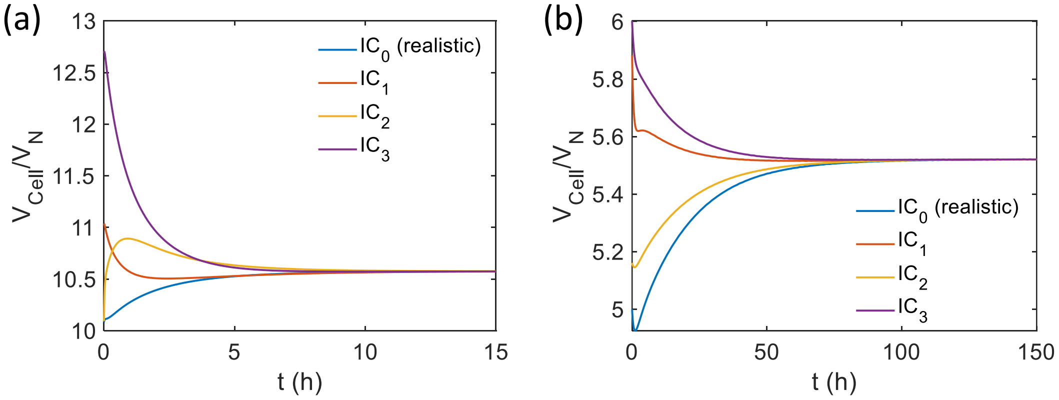

Figure II. (a) Yeast C/N ratio with different initial conditions (IC) (±20% of main text values). (b) Mammalian cell C/N ratio with different IC.

different model parameters (±50% of stated values). In each set of model parameters, the initial conditions vary randomly within ±20%. S1 Fig III shows the C/N ratio with different model parameters. Therefore, numerical results indicate that the C/N ratio is generally stable.

Similarly, we can test the stability of protein and ribosome concentrations (here we take cytoplasmic protein as an example) and the volumetric growth rate (S1 Fig IV). In S1 Fig IVB, the growth rate is calculated as: $\lambda = \frac{1}{V_{Cell}} \frac{dV_{Cell}}{dt}$, where $V_{Cell} = V_C + V_N$ is cell volume.

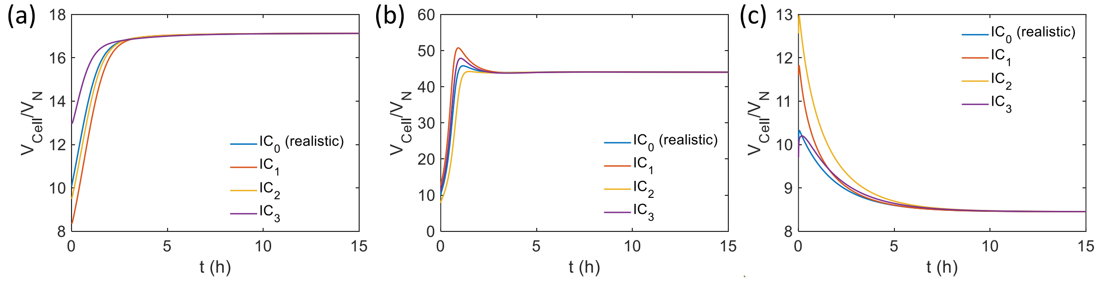

Figure III. Yeast C/N ratio with different parameters and initial conditions. Model parameters are different in (a), (b) and (c), varying ±50%.

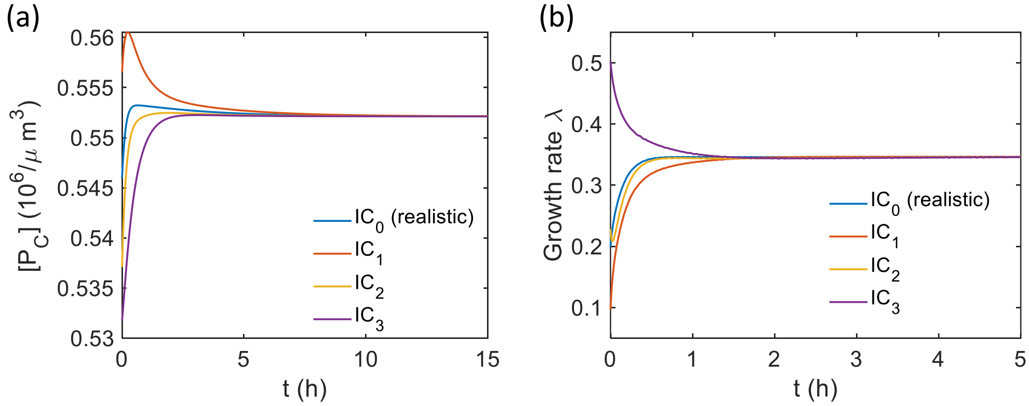

Figure IV. (a) Concentration of cytoplasmic protein. (b) Cell volumetric growth rate. Both quantities are stable when varying initial conditions (IC) within ±20%. In these results, we are using yeast cell parameters from S1 Table B.

Proteome distribution

The governing equations for 2 non-ribosomal protein are:

$$\frac{dA_C}{dt} = \left[t_1 \sum_i P_{C,i} + \sum_i n_{1,i} \frac{d_{1,i} P_{C,i} \sum_i P_{C,i}}{V_C} + n_2 \frac{d_2 R P_C \sum_i P_{C,i}}{V_C}\right] \times \frac{V_C}{V_C + V_N} - \frac{(\sum_i s_{1,i} + s_2) A_C R_C}{V_C}, \tag{11}$$

$$\frac{dP_{C,i}}{dt} = \frac{s_{1,i} A_C R_C}{n_{1i} V_C} - \frac{d_{1,i} P_{C,i} \sum_i P_{C,i}}{V_C} - \left(\frac{t_{4,i} P_{C,i}}{V_C} - \frac{t_{5,i} P_{N,i}}{V_N} \right) \sum_i P_{C,i}, \quad (12)$$

$$\frac{dR_C}{dt} = \frac{t_3 R_N \sum_i P_{C,i}}{V_N} - \frac{d_3 R_C \sum_i P_{C,i}}{V_C} \quad (13)$$

$$\frac{dRP_C}{dt} = \frac{n_3 d_3 R_C \sum_i P_{C,i}}{V_C} + \frac{s_2 A_C R_C}{n_2 V_C} - \frac{t_2 RP_C \sum_i P_{C,i}}{V_C} - \frac{d_2 RP_C \sum_i P_{C,i}}{V_C} \quad (14)$$

$$\frac{dRP_N}{dt} = \frac{t_2 RP_C \sum_i P_{C,i}}{V_C} - s_3 RP_N \quad (15)$$

$$\frac{dR_N}{dt} = \frac{s_3}{n_3} RP_N - \frac{t_3 R_N \sum_i P_{C,i}}{V_N} \quad (16)$$

$$\frac{dP_{N,i}}{dt} = \left(\frac{t_{4,i} P_{C,i}}{V_C} - \frac{t_{5,i} P_{N,i}}{V_N} \right) \sum_i P_{C,i} \quad (17)$$

$$(18)$$

where $P_{C,i}$ is the ith non-ribosomal protein, and $s_{1,i}$ is the corresponding synthesis coefficient. The parameters for the proteome distribution part is shown in S1 Table B:

Table B. Model parameters and their numerical estimates.

Parameter	Description	Values
$n_{1,i}$	Average number of AA in non-ribosomal proteins	400
n_2	Average number of AA in ribosomal proteins	400
n_3	Average number of protein subunits in mature ribosomes	80
t_1	Amino acid import rate coefficient (h^{-1})	400
t_2	Ribosomal protein transport coefficient across nuclear envelop ($\mu m^3/10^6 h$)	9
t_3	Ribosome transport coefficient across nuclear envelop ($\mu m^3/10^6 h$)	1.2
$t_{4,i}$	Non-ribosomal protein transport coefficient into nucleus ($\mu m^3/10^6 h$)	0.8
$t_{5,i}$	Non-ribosomal protein transport coefficient out of nucleus ($\mu m^3/10^6 h$)	0.72
$d_{1C,i}, d_{1N,i}$	Non-ribosomal protein degradation coefficient ($\mu m^3/10^6 h$)	0.1
$d_{2C,i}, d_{2N,i}$	Ribosomal protein degradation coefficient ($\mu m^3/10^6 h$)	0.1
$d_{3C,i}, d_{3N,i}$	Ribosome disassembly coefficient ($\mu m^3/10^6 h$)	0.1
$s_{1,i}$	Non-ribosomal protein synthesis coefficient ($\mu m^3/10^6 h$)	77.5
s_2	Ribosomal protein synthesis coefficient ($\mu m^3/10^6 h$)	125
s_3	Ribosome assembly coefficient (h^{-1})	48
