# Supplementary material for: The correlation between cell and nucleus size is explained by an eukaryotic cell growth model
Source: PLoS Comput Biol. 2022 Feb 18;18(2):e1009400. doi: 10.1371/journal.pcbi.1009400 (PMC8893647; doi:10.1371/journal.pcbi.1009400)
Supplement: Attachment — Submitted filename: Review PCOMPBIO-D-21-01529.pdf [file pcbi.1009400.s002.pdf]

Comments: Cell and Nucleus Volume Growth in Eukaryotic Cells

Overall, the abstract and main goals of this study are very laudable. Indeed, the connectivity been nucleus size and cell growth via the gene expression machinery (i.e. ribosomes) and its connections to cellular transport machinery is quite an interesting point to study. The choice of Yeast and Mammalian cell data as a basis for such a eukaryotic study is quite appropriate. However, I feel that some clarity in the values utilized for each simulation and in the presentation of figures especially of the parameter scans in later figures could be made clearer. In general the parameters for the model should be presented in Tables in the Supporting Information and the equations presented in a code that can be downloaded.

My **major concerns** are summarized below:

Abstract: The authors state that "using RNAseq data, it is possible to construct a detailed proteome distribution". While the authors demonstrate this in theory and via the modeling paradigm used, it would be beneficial to state within the main text that this is not explicitly done in this work using previous RNAseq studies from Yeast or Mammalian cells. Perhaps the authors could state *"Although not explicitly demonstrated in this work, using RNAseq data, it is possible to construct a detailed proteome distribution ..."*

Introduction and Figure 1A: The figure is too small to read the data easily. As many of the earlier papers cited in the introduction 1-3, and especially the 2015 article, mostly consider the nucleus size and shape for cells attached to a surface and when released from the surface following chemical treatment. If the authors have generated the data in Figure 1A, it would be good to have more details about the experiment in the SI.

Modeling Assumptions and Initial Conditions (lines 231-260):

Can you briefly comment on any caveats on scaling up from Yeast growth related parameters to achieve initial conditions for Mammalian cells? Are the values given in Table 2 from the literature used or the re-scaled values. How different they are would also be interesting. Growth media?

Does the model truly half the amounts of cellular resources at each cell division event or does it restart each cell with the initial conditions of the first cell that is simulated?

Initial Conditions for Simulations:
How do the initial conditions compare to the values in Earnest et al JCPB 2017: 5x10^7 proteins, 180K ribosomes, …. How where the values selected in this work?: 6x10^7 proteins, 150K ribosome --- *The citations for the values presented in Earnest et al JCPB for Yeast are as follows: von der Haar et al BMC. Syst. Biol. 2008, Futcher et al Mol. Cell. Biol. 1999.*

Figure 4: Is the difference in the behavior of the increases in Cell Volume for the quiescence and poor amino acid cases in Yeast and HeLa cells primarily due to the difference in volume

(HeLa > Yeast) between the two cells? If so, should this be stated in the text or is it more due to "the balance between protein synthesis and degradation" as you state in **line 330**?

Figure 5 - Heatmap vs Contour maps: It may be clearer if this was made a 2D heatmap like *Fig. 6* and if X's or markers were placed on the exact conditions used in simulations (and Reported in *Table 2*) that are most appropriate.

Figure 5: Out of curiosity, does CellV and doubling time ever plateau when increasing t1 (the amino acid transport rate, it does not seem that it does) or does CellV continue to increase for Yeast (as is shown in *Fig. 5a and Fig.5f*) with increasing t1 via your model parameters?

Figure 8: This figure could be made clearer in that *Fig8a-c* and *Fig8d-f* respectively could benefit from having the same axes scales (which they do not, these seem to vary from 0.6-0.8 for the same quantity, i.e. t1), which would make a 1-to-1 comparison of growth conditions more amenable.

Lines 487-540: It is good that the authors eventually discuss the stochastic nature of gene expression and some of its effects on the cell cycle. I would like to have seen this mentioned earlier, perhaps in the introduction. It would also be good to discuss and cite some other work on stochastic gene expression, its impacts on various parts of the cell cycle, and combinations of analytic/deterministic processes with stochastic processes. Beside the early work of Elowitz on bacteria and Arkin, it would be good to mention both the existing analytical and computational studies. For example, the work of Ramon Grima in comparing analytic and stochastic simulations in a eukaryotic cell to obtain protein numbers (Cao and Grima, PNAS, 2020) could be an interesting discussion or comparison to make to this model. Additionally, of interest are the simulations ribosome biogenesis stochastically in a dividing E. coli cell (Earnest et al., Biopolymers, 2016) or the hybrid stochastic-deterministic simulation technique (Bianchi et al., IET Systems Biology, 2018) applied to the galactose switch in yeast.

Discussion and Conclusions: Where you state "amino acid import coefficient t1 is more important for exponentially growing cells" in **line 574** would be an opportune place in which to address my earlier question about the relationship between t1 and Volume Growth measured in *Figure 5*.

Minor

1.Where you state, "stochastic dynamical systems response to changes in environmental systems" in **line 585** it should read "**respond** to changes". Also, a relevant citation for this statement would be: Wolf et al *eLife* 2015 among others.
2. **line** 305-307: Indicate whether replication initiation corresponds to DNA replication initiation or cell division initiation, as this could be unclear to readers less familiar with the budding process of yeast.

308: “The division mechanism our simulation does not affect” sounds like there is a word missing in the thought.
