# Supplementary material for: The correlation between cell and nucleus size is explained by an eukaryotic cell growth model
Source: PLoS Comput Biol. 2022 Feb 18;18(2):e1009400. doi: 10.1371/journal.pcbi.1009400 (PMC8893647; doi:10.1371/journal.pcbi.1009400)
Supplement: Attachment — Submitted filename: rep.pdf [file pcbi.1009400.s003.pdf]

In this paper, the authors tackle an interesting problem of theoretically explaining the observed proportionality between Cellular and Nuclear volume, across various Eukaryotic cells and growth conditions. To explain this observed proportionality, the authors develop a simple, coarse-grained mathematical model of protein and ribosome synthesis. Apart from synthesis, the authors also extensively study the effect of transport, assembly, disassembly, and degradation, on the cellular growth rate and the above-mentioned proportionality, although still at a coarse grained level.

While the modeling of most of these processes may not be novel, the modeling of transport across nuclear membrane and the theoretical study of the Cytoplasm to Nucleoplasm volume ratio (C/N) seems novel to me. The paper is well written and well presented in most places. However, there is potential to significantly improve the paper and its presentation. Below are my concerns and suggestions:

1. The most important issue for me is the omission of the volume of the DNA in the calculation of nuclear volume. The authors, in line 222, mention that the weight of DNA can be neglected, but also agree in line 221 that its volume is not negligible. Therefore, it is surprising that they did not account for the DNA's volume in calculating the nuclear volume. It should not be very difficult to incorporate it, as the DNA content of the cell is also expected to grow at the same rate as other proteins in balanced growth conditions. It would add to the nucleoplasm volume proportionally, thereby maintaining the constancy of the ratio.

2. This brings me to my second point, on the lack of a clear discussion on balanced growth (they do briefly mention it in line 15-16, and indirectly in the lines 40-42, and 311-313). The authors would agree that most of the findings in the paper can be understood under the concept of balanced growth, which is well appreciated by now in the realm of prokaryotic cells (see [1, 2] for example). Therefore, a bit more expanded discussion on this topic in the introduction will give the readers a clearer picture.

3. The authors claim that the cell may be optimizing a set of parameters to regulate the growth rate and C/N ratio (in abstract and section on page 16 for example). While it is easy to understand why the cell would want to control its growth rate, it is not clear why it would want to directly control the C/N ratio. If my understanding is correct, any level of nuclear membrane permeability will give rise to balanced growth. Optimal permeability is only needed to ensure that all the components are available across the membrane in the right quantities, so as to maximize the growth rate. Therefore, I would think that the C/N ratio is a passive outcome of regulating the growth rate in a given situation. The authors should clarify this.

4. It is unclear in the beginning whether the paper is going to explain experimental results or study the model purely theoretically. While it gives the impression that it will

do the former, there is no comparison with experiments (especially in Fig.4). Indeed, all the subsequent figures are from the model only. Therefore, it will be helpful if the authors clarify the scope of the paper a bit more at the outset.

5. In line 328, the authors say that their model predicts linear growth in poor nutrient medium. Is it only approximately linear, as in being transitionary between exponentially growing and stationary states, or can this linearity be demonstrated analytically for some particular nutrient concentration? The authors should elaborate on this.

6. The effect of t_2 on C/N (line 384) comes from the assumption that the ribosome takes the same volume as an individual ribosomal protein. Given that 80 ribosomal proteins go into forming a ribosome, this assumption does not seem correct. This assumption also influences much of the later analysis in the paper.

Minor issues:

1. In line 14, N/C, or C/N abbreviation should be defined before using it.

2. In line 52, it should be Fig. 1(c) and not 1(b). This is also the case in line 98 and line 144.

3. Naming the parameter s_{1R} as s_2, and s_2 as s_3 would have made the nomenclature consistent with the numbering for other parametrs like n and d, thereby making it easier to remember.

4. Fig. 2 should be considerably improved, to show steps like codons in mRNA on which ribosomes move, and amino acid arrival. Otherwise it should be completely removed. In its current form it does not teach much.

5. The authors should provide more details on the algorithm/software used to simulate the system of equations.

6. In line 321, references 35 and 36 are for enhanced degradation in stem cells. The authors should provide reference for enhanced degradation in yeast and HeLa cells, or discuss why they expect it to be true in these cells as well.

7. The authors should mention if same or different values of cytoplasm-nucleoplasm transport coefficients were used in the three cases of Fig.4.

8. In line 350, it should be (k)-(o) and not (k)-(l).

9. In line 356, it is not clear why increase in t_2 should decrease growth rate. The au-

thors should explain it a bit more. I would think that increase in transport of ribosomal protein to nucleus will only enhance ribosome assembly, and can only increase growth rate. Decrease in cytoplasmic ribosomal protein should not reduce the growth rate in their model if the number of mature ribosomes are same.

10. The section on protein distribution in individual cells (last section of results) is beyond the central message of this paper and removing that section will improve the sharpness of the central message of the paper.

11. Reference/s should be added after the sentence in line 582. Although I feel that this whole paragraph is not necessary for the storyline of the paper.

References

[1] Pandey, P. P., Singh, H., Jain, S. (2020). Exponential trajectories, cell size fluctuations, and the adder property in bacteria follow from simple chemical dynamics and division control. Physical Review E, 101(6), 062406.

[2] Roy, A., Goberman, D., Pugatch, R. (2021). A unifying autocatalytic network-based framework for bacterial growth laws. PNAS, 118(33).
