# Supplementary material for: The correlation between cell and nucleus size is explained by an eukaryotic cell growth model
Source: PLoS Comput Biol. 2022 Feb 18;18(2):e1009400. doi: 10.1371/journal.pcbi.1009400 (PMC8893647; doi:10.1371/journal.pcbi.1009400)
Supplement: Attachment — Submitted filename: Response Letter_PCB_03.docx [file pcbi.1009400.s004.docx]

**Response to Reviewer #1:**

***Comment #1:***

*The work "The Correlation Between Cell and Nucleus Size is Explained by a Eukaryotic Cell Growth Model" used a computational model to quantify factors influencing the cell volume to nuclear volume ratio (C/N ratio). While the regulatory mechanism of C/N ratio is of scientific importance and this work exhibit how simple models could explain complex biological phenomena, several major issues should be resolved before publishing this work as a formal academic paper.*

**Response:** We thank the reviewer for the comment.

***Comment #2:***

*This work heavily relies on simulation, while more quantitative insights on C/N ratio could be obtained by analytical approximations. The major component of this work, Eq. 23-29. is in the unit of [number per cell], instead of concentration. Once they are changed into the unit of concentration, one can work towards the steady-state solution. This could be done by dividing a "number" quantity X by their corresponding volumes V, changing into a concentration quantity x: if dX/dt=F(X), d(X/V)/dt=(1/V)*dX/dt-(X/V)*(1/V)*dV/dt as the increasing speed of volume relative to the current volume, (1/V)*dV/dt, can be viewed as the growth rate g, one could obtain a differential equation of the concentration x, with an growth-induced dilution term: d(X/V)/dt=dx/dt=(1/V)*F(X)-x*g; for nucleus-related term, it is g_N; for cytoplasm-related term, it is g_C;*

*b. With the growth-induced dilution term, Eq. 23-29, when changed into the concentration format, should yield a steady-state solution about the concentration of proteins and ribosomes in the nucleus and the cytoplasm. Under this work's assumption of "cell volume is proportional to the protein and macromolecular number" (which itself needs more support from previous researches), a constant C/N ratio would be an intuitive result of "equilibrium between in/out nucleus transportation and diffusion". Then one can investigate how parameters influencing transportation and diffusion influence the relation between g_C and g_N, and the stability of C/N ratio.*

**Response:** We thank the reviewer for the comment. We have performed some analytical approximation following the reviewer’s suggestion. After changing the unit into concentration and neglecting the degradation terms, denoting the cytoplasm to nucleus volume ration ($V_{Cyto}/V_{N}$) to be $q$, the equations are written as:

$$\left\{ \begin{aligned} \frac{d[A_{C}]}{dt}=\frac{t_{1}\left[ P_{C} \right]}{1+\frac{1}{q}}-(s_{1}+s_{2})\left[ A_{C} \right]\left[ R_{C} \right]-\left[ A_{C} \right]g_{C} \\ \frac{d\left[ P_{C} \right]}{dt}=\frac{s_{1}}{n_{1}}\left[ A_{C} \right]\left[ R_{C} \right]-\left( t_{4}\left[ P_{C} \right]-t_{5}\left[ P_{N} \right] \right)\left[ P_{C} \right]-\left[ P_{C} \right]g_{C} \\ \frac{d\left[ R_{C} \right]}{dt}=t_{3}\left[ R_{N} \right]\left[ P_{C} \right]-\left[ R_{C} \right]g_{C} \\ \frac{d\left[ RP_{C} \right]}{dt}=\frac{s_{2}}{n_{2}}\left[ A_{C} \right]\left[ R_{C} \right]-t_{2}\left[ RP_{C} \right]\left[ P_{C} \right]-\left[ RP_{C} \right]g_{C} \\ \frac{d\left[ RP_{N} \right]}{dt}=t_{2}\left[ RP_{C} \right]\left[ P_{C} \right]q-s_{3}\left[ RP_{N} \right]-\left[ RP_{N} \right]g_{N} \\ \frac{d\left[ R_{N} \right]}{dt}=\frac{s_{3}}{n_{3}}\left[ RP_{N} \right]-t_{3}\left[ R_{N} \right]\left[ P_{C} \right]q-\left[ R_{N} \right]g_{N} \\ \frac{d\left[ P_{N} \right]}{dt}=\left( t_{4}\left[ P_{C} \right]-t_{5}\left[ P_{N} \right] \right)\left[ P_{C} \right]q-\left[ P_{N} \right]g_{N} \end{aligned} \right.$$

With volume equation:

$$\left\{ \begin{aligned} V_{Cyto}=(P_{C}+RP_{C}+R_{C})/r_{1} \\ V_{N}=(P_{N}+RP_{N}+R_{N})/r_{2} \end{aligned} \right.$$

In steady state, the left hand side is set to 0. Since the final solution for all the molecule number can be written as $\boldsymbol{C}_{\boldsymbol{7\times1}}e^{\lambda t}$, we can express growth rate as $g_{C}=g_{N}=\lambda$. However, since the equation set has 7 dimensions and nonlinearly coupled, it is difficult to analyze the dependence of $\lambda$ and $V_{Cell}/V_{N}$ on different parameters based on this equation.

To obtain analytical results, we made the following simplifications: (1) assume amino acid is abundant and do not consider the amino acid dynamics. The protein synthesis rate is only proportional to ribosome number. (2) Nuclear ribosome $R_{N}$ is much less than cytoplasmic ribosome number $R_{C}$ (We denote the total number of ribosomes to be $R$ so $R_{C}=R$, $R_{N}\sim0$). (3) Transport rate of ribosomal protein is constant ($t_{2}$). (4) We only consider the net import of non-ribosomal proteins and the rate is set to be proportional to cytoplasmic non-ribosomal protein number $P_{C}$. Under these assumptions, we can get:

$$\left\{ \begin{aligned} \frac{dP_{C}}{dt}=s_{1}R-t_{4}P_{C} \\ \frac{dR}{dt}=\frac{s_{3}}{n_{3}}RP_{N} \\ \frac{dRP_{C}}{dt}=s_{2}R-t_{2} \\ \frac{dRP_{N}}{dt}=t_{2}-s_{3}RP_{N} \\ \frac{dP_{N}}{dt}=t_{4}P_{C} \end{aligned} \right.$$

These equations can be directly solved as:

$$\left\{ \begin{aligned} P_{C}=C_{4}e^{-t_{4}t}+s_{1}\left[ \frac{C_{1}}{n_{3}\left( t_{4}-s_{3} \right)}e^{-s_{3}t}+\frac{C_{2}}{t_{4}}+\frac{t_{2}}{n_{3}t_{4}}\left( t-\frac{1}{t_{4}} \right) \right] \\ R=\frac{C_{1}}{n_{3}}e^{-s_{3}t}+\frac{t_{2}}{n_{3}}t+C_{2} \\ RP_{C}=-\frac{C_{1}s_{2}}{n_{3}s_{3}}e^{-s_{3}t}+\frac{t_{2}s_{2}}{2n_{3}}t^{2}+\left( C_{2}s_{2}-t_{2} \right)t+C_{3} \\ RP_{N}=-C_{1}e^{-s_{3}t}+\frac{t_{2}}{s_{3}} \\ P_{N}=-C_{4}e^{-t_{4}t}-\frac{s_{1}C_{1}t_{4}}{n_{3}\left( t_{4}-s_{3} \right)s_{3}}e^{-s_{2}t}+s_{1}\left( C_{2}-\frac{t_{2}}{n_{3}t_{4}} \right)t+\frac{s_{1}t_{2}}{2n_{3}}t^{2}+C_{5} \end{aligned} \right.$$

Where $C_{1}$~$C_{5}$ are integral constant.

The result here shows that the growth is basically proportional to $t^{2}$, which doesn’t capture the feature of exponential growth. We can also get the steady state $\frac{V_{Cell}}{V_{N}}=1+\frac{s_{2}}{s_{1}}$, under the assumption that volume coefficients are the same for nucleus and cytoplasm ($r_{1}=r_{2}$). This means the steady state $\frac{V_{Cell}}{V_{N}}$ decreases with $s_{1}$ and increases with $s_{2}$, which basically agrees with our numerical result of yeast cell in Fig. 4 (j). However, this result doesn’t reflect the influence of ribosome synthesis and transport on C/N ratio, which can be more important. Therefore, we don’t think the analytical approximation can capture the major features of our model.

***Comment #3:***

*The current manuscript lacks a clear emphasis on "which scientific questions were solved by this work". While C/N ratio is a field of interest by many researchers (which is also not yet sufficiently reviewed in this current manuscript), is the current manuscript focusing on how a constant C/N ratio can be maintained, or which biological processes may influence this ratio? What are the model results that are supported by previous observations, and what are the unexpected findings that inspire further investigation? For example, this work suggested "for the nutrient-limited cell, the C/N ratio fluctuates periodically", and " s1 and s1r have opposite effects on the C/N ratio" has that been observed in previous experiments?*

**Response:** We thank the reviewer for the comment. The main question we’d like to answer is why cell volume is proportional to the nuclear volume, and how different kinetic parameters influence the C/N ratio and growth rate. We propose a mechanistic explanation of C/N ratio, and obtain insights on the stability of C/N ratio and its sensitivity to different parameters. To make this point clear, we have added some description in the first paragraph in introduction.

Unfortunately, experiments have not systematically studied these questions, but experiments can potentially test our model predictions.

***Comment #4:***

*Other than how different parameters influences C/N ratio, the format of equations should also be discussed: Which of the three processes, synthesis/transport/degradation, or subprocesses, plays essential roles in keeping the C/N ratio stable? It seems that the transportation and diffusion between the nucleus and cytoplasm are sufficient to maintain the C/N ratio stable, then what are the roles played by ribosome and protein synthesis? Is the degradation part necessary in this model?*

**Response:** We thank the reviewer for the comment. These questions are explored in FIG.5 and FIG. 8. It is easy to see that the C/N ratio is mostly sensitive to protein transport rate across nuclear envelop (t_4_ and t_5_). In other words, these two parameters are most important in keeping C/N ratio stable. Synthesis of ribosomes and proteins do influence the C/N ratio (*e.g.*, increase of s_2_ slightly increases the C/N ratio), but ultimately, synthesized products will be distributed proportionally in nucleus and cytoplasm, thus having little influence compared to protein transport across nuclear envelop.

The degradation part contains both protein degradation and ribosome disassembly. There could be some non-monotonic influence on growth rate and C/N ratio, as is shown in FIG.7. Also, degradation will also influence the protein to ribosome ratio, and non-ribosomal/ ribosomal protein ratio. Degradation is also an important part in quiescent cells because there is increased degradation in quiescent cells for both yeast and mammalian cells

***Comment #5:*** *Also, does the assumed format of equations influence the modeling results? Some assumptions in this manuscript are different from previous works: This work assumes a linear relationship between protein synthesis rate and the amino acid concentration, both in nutrient-rich and nutrient-limited conditions, which are different from the Michaelis–Menten format in Scott's 2014 work about the growth law; Also, the square relationship between protein number and its degradation rate is different from the linear degradation term in most modeling works. These differences should be supported, and whether they influence the modeling results should be discussed.*

**Response:** We thank the reviewer for the comment. In Scott 2014, the Michaelis–Menten form is applied to the amino acid import efficiency (which corresponds to $t_{1}$ in our model). However, for the protein synthesis rate, they did not directly connect it to intracellular amino acid concentration. In the steady state when intracellular amino acid concentration does not change, the growth rate (or protein synthesis rate) is proportional to $t_{1}$ (transport efficiency), which takes the Michaelis–Menten format as a function of extracellular amino acid concentration, instead of intracellular amino acid concentration. However, we are assuming the protein synthesis rate is proportional to intracellular AA concentration. The extracellular concentration is incorporated in $t_{1}$ in our model. Therefore, there is no contradiction between our assumption and the assumption in Scott’s work. In our model, we assume the extracellular nutrient environment only directly influences the amino acid transport and this influence is different for nutrient rich and nutrient-limited cases. Amino acid import will influence the intracellular amino acid concentration, which will further influence protein synthesis. Therefore, the final growth is different for nutrient-rich and limited case.

Also, in our model, the rich-nutrient case corresponds to the high amino acid concentration limit in Michaelis–Menten form, where $t_{1}$ is a constant. The poor-nutrient case we are considering is a nutrient-limited but transporter-sufficient case, which is not considered in Scott’s work in 2014. We added a few sentences to discuss this in the paper.

The reason why there’s a squared relationship is that we assume the degradation rate is not only proportional to the number of protein to be degraded but also protein assisting degradation (e.g., protein in lysosome, proteasome). Since we assume both the protein numbers are proportional to total protein number, there should be a square term. We do have a linear form in quiescent case, with the assumption that the lysosomes and proteasomes assisting degradation are sufficient. In such case, the degradation rate is only proportional to the concentration of protein to be degraded.

This brings us to an important point about the model of Scott 2014, and our current work. When modeling bi-molecular (or higher order) reactions, the rate of reaction is proportional to the concentration of reactants, which necessarily involves a volume factor. Scott 2014 models changes in cell mass, which is ok for uni-molecular type of kinetics. But since protein synthesis and degradation are presumably bi-molecular reactions, any models of cell growth must necessarily involve cell volume, which determines the concentrations of reactants.

***Comment #6:***

*Other issues include:*

*Formatting: Eq 22-23 extends outside of the page limit. Eq. 23-29 has no labeling.*

*In line 285, "ribosome disassembly and protein degradation rates in rapidly growing cells are 1/10 of the synthesis rate" is pretty arbitrary. The degradation rate can be estimated by the protein synthesis rate and the cell mass.*

*In Fig.4 C, the decrease of cell volume in quiescent status into near-zero value within 2 hours is not very realistic. Is that due to the assumed form of degradation?*

**Response:** We thank the reviewer for the comment. We have addressed the formatting issue in Eq 22-23.

For the degradation part, the degradation rate can vary a lot for different proteins [1]. For example, in yeast, the degradation rate constant could vary from 0.005 – 1 (e^-5^~e^0^) for different proteins in the first order decay estimation, which corresponds to the form: $\frac{dP}{dt}=-kP$, where *k* is the degradation rate. Comparing with our model: $\frac{dP}{dt}\sim-\frac{d\cdot P}{V}P$ (approximation when all the degradation rates are the same), where P~15 million and V~30${\mu m}^{3}$ we can get the range of our degradation rate coefficient: 0.01~2 ($\frac{{\mu m}^{3}}{10^{6}h}$). We assume mammalian cells have the same range of degradation coefficient. Our assumption (*i.e.* degradation are 1/10 of synthesis) gives the degradation coefficients as: 0.1 and 0.01 for yeast and mammalian cell, respectively. These values are in the reasonable range. We have added more explanation in subsection “Kinetic parameters”.

In FIG 4c, the y axis limit is around 30-60, and the cell volume reaches a constant around 30 (${\mu m}^{3}$) in quiescent case, which is a reasonable result for stationary cell.

**Response to Reviewer #2:**

***Comment #1:***

*Overall, the abstract and main goals of this study are very laudable. Indeed, the connectivity been nucleus size and cell growth via the gene expression machinery (i.e. ribosomes) and its connections to cellular transport machinery is quite an interesting point to study. The choice of Yeast and Mammalian cell data as a basis for such a eukaryotic study is quite appropriate. However, I feel that some clarity in the values utilized for each simulation and in the presentation of figures especially of the parameter scans in later figures could be made clearer. In general the parameters for the model should be presented in Tables in the Supporting Information and the equations presented in a code that can be downloaded*

**Response:** We thank the reviewer for the positive comment and constructive suggestion. We have clarified values used in simulation in each figure caption and presented all parameters in table in the manuscript. We have also provided the MATLAB code we used for simulation.

***Comment #2:***

***Abstract:*** *The authors state that "using RNAseq data, it is possible to construct a detailed proteome distribution". While the authors demonstrate this in theory and via the modeling paradigm used, it would be beneficial to state within the main text that this is not explicitly done in this work using previous RNAseq studies from Yeast or Mammalian cells. Perhaps the authors could state "Although not explicitly demonstrated in this work, using RNAseq data, it is possible to construct a detailed proteome distribution ...”*

**Response:** We thank the reviewer for the comment. We have revised the corresponding part following the reviewer’s suggestion.

***Comment #3:***

***Introduction and Figure 1A:*** *The figure is too small to read the data easily. As many of the earlier papers cited in the introduction 1-3, and especially the 2015 article, mostly consider the nucleus size and shape for cells attached to a surface and when released from the surface following chemical treatment. If the authors have generated the data in Figure 1A, it would be good to have more details about the experiment in the SI.*

**Response:** We thank the reviewer for the comment. We have enlarged Fig.1a to make it easier to read. We have also added details about the experiment related to Fig 1 in the SI.

***Comment #4:***

***Modeling Assumptions and Initial Conditions (lines 231-260):*** *Can you briefly comment on any caveats on scaling up from Yeast growth related parameters to achieve initial conditions for Mammalian cells? Are the values given in Table 2 from the literature used or the re-scaled values. How different they are would also be interesting. Growth media? Does the model truly half the amounts of cellular resources at each cell division event or does it restart each cell with the initial conditions of the first cell that is simulated?*

**Response:** We thank the reviewer for the questions. In our simulation, most of the initial conditions for mammalian cells are based on literature. Only the ratio of non-ribosomal number to ribosomal protein number ($P/RP$) is assumed to be the same as yeast cell.

Most of the values given in Table 2 are based on indirect estimation from data in literature for both yeast and mammalian cells. Only the transport rates of ribosomal protein and ribosome ($t_{2}$, $t_{3}$) for mammalian cells are rescaled based on yeast data because these data for mammalian cells are hard to find in literature. The scaling assumption is due to the fact that the transport rate (number/min) is proportional to cell volume and inversely proportional to cell cycle time. To compare literature-based data and scaled data, we recalculate mammalian cell parameters (except transport rates of ribosomal protein and ribosome) by scaling up from yeast cells and compare them with the literature-based estimation, as is shown in the supporting information. Only ribosome synthesis coefficient ($s_{3}$) and amino acid transport coefficient ($t_{1}$) are slightly different from literature-based values, while others are the same. The similarity and difference between the two sets of parameters can be explained as follows: The protein concentration of yeast cells is the same as that of mammalian cells, therefore scaling up the protein-related parameters from yeast gives the same results as the literature-based results. However, the ribosome concentration and amino acid concentration is higher in yeast than mammalian cells, and this explains why the rescaled values are larger than the literature-based values. To clarify this, we add some discussion in section “Parameter estimation”.

In the simulation, we half the amounts of cellular resources at each cell division event instead of restarting with initial conditions. We have changed the relevant description to make this point clear.

***Comment #5:***

***Initial Conditions for Simulations:*** *How do the initial conditions compare to the values in Earnest et al JCPB 2017: 5x10^7 proteins, 180K ribosomes, …. How where the values selected in this work?: 6x10^7 proteins, 150K ribosome --- The citations for the values presented in Earnest et al JCPB for Yeast are as follows: von der Haar et al BMC. Syst. Biol. 2008, Futcher et al Mol. Cell. Biol. 1999.*

**Response:** We thank the reviewer for the comment. In our case, the protein number and ribosome number in new born cells are ~2.7$\times$10^7^ and 150K, respectively, which are similar to the values in Earnest *et al* JCPB 2017 [2]. The sources for the data are shown in Table 1 and Table 2. The details of estimation can be found in “Parameter estimation” part. We also cite Earnest et al JCPB 2017 for comparison with our parameter values.

***Comment #6:***

***Figure 4:*** *Is the difference in the behavior of the increases in Cell Volume for the quiescence and poor amino acid cases in Yeast and HeLa cells primarily due to the difference in volume (HeLa > Yeast) between the two cells? If so, should this be stated in the text or is it more due to "the balance between protein synthesis and degradation" as you state in line 330?*

**Response:** We thank the reviewer for the question. The difference between the behaviors in yeast and HeLa cells are primarily due to the difference in relative values of the kinetic parameters and initial conditions (e.g. the ribosome assembly coefficient *s_2_* for mammalian cells is smaller than that of yeast cells; the relative amount of amino acid in mammalian cell is less than yeast cell). The “balance between protein synthesis and degradation” leads to the final constant volume in quiescent case, but how this steady state is achieved is determined by the relative values of the kinetic parameters, which are different for yeast and mammalian cells.

***Comment #7:***

***Figure 5:*** *Heatmap vs Contour maps: It may be clearer if this was made a 2D heatmap like Fig. 6 and if X's or markers were placed on the exact conditions used in simulations (and Reported in Table 2) that are most appropriate.*

*Out of curiosity, does CellV and doubling time ever plateau when increasing t1 (the amino acid transport rate, it does not seem that it does) or does CellV continue to increase for Yeast (as is shown in Fig. 5a and Fig.5f) with increasing t1 via your model parameters?*

**Response:** We thank the reviewer for the suggestion. We have followed the suggestion and remade the plot in 2D heatmap and added markers on the exact values we used in simulation.

FIG.5 (a) and (f) show how the growth rate is influenced by various parameters. To answer whether the growth rate can reach plateau when increasing *t*_1_, we did the parameter scan in a larger range, as is shown below (Fig (a) is for yeast cell and Fig (b) is for HeLa cell). The result shows that for both cells, the growth rate doesn’t plateau with increase of *t*_1_. In reality, however, there should be an upper limit for amino acid transport rate (*t*_1_). To make this point clear, we have added some discussion in subsection “Effects of protein transport and synthesis on cell growth rate”.

***Comment #8:***

***Figure 8:*** *This figure could be made clearer in that Fig8a-c and Fig8d-f respectively could benefit from having the same axes scales (which they do not, these seem to vary from 0.6-0.8 for the same quantity, i.e. t1), which would make a 1-to-1 comparison of growth conditions more amenable.*

**Response:** We thank the reviewer for the suggestion. We have revised the figures so that they have the same axis scales.

***Comment #9:***

***Lines 487-540:*** *It is good that the authors eventually discuss the stochastic nature of gene expression and some of its effects on the cell cycle. I would like to have seen this mentioned earlier, perhaps in the introduction. It would also be good to discuss and cite some other work on stochastic gene expression, its impacts on various parts of the cell cycle, and combinations of analytic/deterministic processes with stochastic processes. Beside the early work of Elowitz on bacteria and Arkin, it would be good to mention both the existing analytical and computational studies. For example, the work of Ramon Grima in comparing analytic and stochastic simulations in a eukaryotic cell to obtain protein numbers (Cao and Grima, PNAS, 2020) could be an interesting discussion or comparison to make to this model. Additionally, of interest are the simulations ribosome biogenesis stochastically in a dividing E. coli cell (Earnest et al., Biopolymers, 2016) or the hybrid stochastic-deterministic simulation technique (Bianchi et al., IET Systems Biology, 2018) applied to the galactose switch in yeast.*

**Response:** We thank the reviewer for the comment. We have added some discussion and citations about the stochastic gene expression in introduction. Stochastic effects are not important for our model because we are modeling total populations of protein and ribosomes, whose numbers are larger, and therefore fluctuations should be negligible. For individual genes, stochastic effects are of course important, and we have added these citations.

***Comment #10:***

***Discussion and Conclusions:*** *Where you state "amino acid import coefficient t1 is more important for exponentially growing cells" in line 574 would be an opportune place in which to address my earlier question about the relationship between t1 and Volume Growth measured in Figure 5.*

**Response:** We thank the reviewer for the suggestion. We have added some discussion in subsection “Effects of protein transport and synthesis on cell growth rate” to address the reviewer’s previous question.

***Comment #11:***

***Minor:*** *1. Where you state, "stochastic dynamical systems response to changes in environmental systems" in line 585 it should read "respond to changes". Also, a relevant citation for this statement would be: Wolf et al eLife 2015 among others.*

*2. line 305-307: Indicate whether replication initiation corresponds to DNA replication initiation or cell division initiation, as this could be unclear to readers less familiar with the budding process of yeast.*

*308: “The division mechanism our simulation does not affect” sounds like there is a word missing in the thought*

**Response:** We thank the reviewer for the comment. We have corrected the grammar mistakes and added the citation in line 585. The replication initiation means cell division initiation. We have modified the description to make it clearer.

**Response to Reviewer #3:**

***Comment #1:***

*In this paper, the authors tackle an interesting problem of theoretically explaining the*

*observed proportionality between Cellular and Nuclear volume, across various Eukaryotic*

*cells and growth conditions. To explain this observed proportionality, the authors develop a*

*simple, coarse-grained mathematical model of protein and ribosome synthesis. Apart from*

*synthesis, the authors also extensively study the effect of transport, assembly, disassembly,*

*and degradation, on the cellular growth rate and the above-mentioned proportionality,*

*although still at a coarse grained level.*

*While the modeling of most of these processes may not be novel, the modeling of trans-*

*port across nuclear membrane and the theoretical study of the Cytoplasm to Nucleoplasm*

*volume ratio (C/N) seems novel to me. The paper is well written and well presented in*

*most places. However, there is potential to significantly improve the paper and its presentation.*

**Response:** We thank the reviewer for the summary and positive comments.

***Comment #2:***

*The most important issue for me is the omission of the volume of the DNA in the calculation of nuclear volume. The authors, in line 222, mention that the weight of DNA can be neglected, but also agree in line 221 that its volume is not negligible. Therefore, it is surprising that they did not account for the DNA’s volume in calculating the nuclear volume. It should not be very difficult to incorporate it, as the DNA content of the cell is also expected to grow at the same rate as other proteins in balanced growth conditions. It would add to the nucleoplasm volume proportionally, thereby maintaining the constancy of the ratio.*

**Response:** We thank the reviewer for the comment. The reason why we neglect DNA is as follows: In our model, the nuclear volume is determined by the assumption that the nucleus maintains a constant osmotic pressure. In this case, the nuclear volume depends only on the molecular number, and is independent of volume occupied by molecules, as described in Part: “*On the Mapping Between Molecular Content and Cell and Nucleus Size*”. DNA has more molecular weight (~650kDa for a 1000 bp DNA strand) than protein (40kDa). However, the total mass of DNA (~6 pg [3]) is negligible compared to proteins and ribosomes (~150pg in total and ~30pg in nucleus [4]), and therefore the osmotic pressure contribution from DNA should be small. Therefore, we neglect the contribution of DNA to nucleus volume. Furthermore, from Fig. 1a, is can be seen that the nuclear size continuously expands throughout the cell cycle, but DNA only doubles in the S and M phases. Therefore, there is no visible signature of DNA increase. To make this point clear, we have modified the corresponding part in subsection “On the Mapping Between Molecular Content and Cell and Nucleus Size”.

***Comment #3:***

*This brings me to my second point, on the lack of a clear discussion on balanced growth (they do briefly mention it in line 15-16, and indirectly in the lines 40-42, and 311- 313). The authors would agree that most of the findings in the paper can be understood under the concept of balanced growth, which is well appreciated by now in the realm of prokaryotic cells (see [1, 2] for example). Therefore, a bit more expanded discussion on this topic in the introduction will give the readers a clearer picture.*

**Response:** We thank the reviewer for the comment. There are two concepts of balance in this paper. In line 15-16, “A balanced proteome is necessary to achieve optimal growth control” means that the right ratio between different components of the proteome (e.g., ratio between ribosomal and non-ribosomal protein) is necessary to reach the maximum overall cell growth rate. This “balance” refers to proteome balance.

However, the “balance” the reviewer mentions is the fact that different compartments in cell grow proportionally. This is due to the balance between the net nuclear import and the cellular uptake and synthesis.

To clarify this point, we have expanded more on balanced growth in the introduction part.

***Comment #4:***

*The authors claim that the cell may be optimizing a set of parameters to regulate the growth rate and C/N ratio (in abstract and section on page 16 for example). While it is easy to understand why the cell would want to control its growth rate, it is not clear why it would want to directly control the C/N ratio. If my understanding is correct, any level of nuclear membrane permeability will give rise to balanced growth. Optimal permeability is only needed to ensure that all the components are available across the membrane in the right quantities, so as to maximize the growth rate. Therefore, I would think that the C/N ratio is a passive outcome of regulating the growth rate in a given situation. The authors should clarify this.*

**Response:** We thank the reviewer for the comment. In our opinion, regulations of both growth rate and C/N ratio are important. The relative nucleus size plays important roles in important processes such as cell migration [5] and genome regulation [6]. During migration, the cell nucleus can deform and the nuclear size influences the migration speed. At the nuclear periphery, chromatins are associated with the nuclear lamina through lamina-associated domains (LADs), which aids functional organization of the genome. This process may also be related to the relative nuclear size through mechanical connections.

To clarify this, we modified the corresponding part in subsection “Optimal strategies for regulating cell growth and C/N ratio” in the manuscript as:

***Comment #5:***

*It is unclear in the beginning whether the paper is going to explain experimental results or study the model purely theoretically. While it gives the impression that it will do the former, there is no comparison with experiments (especially in Fig.4). Indeed, all the subsequent figures are from the model only. Therefore, it will be helpful if the authors clarify the scope of the paper a bit more at the outset.*

**Response:** We thank the reviewer for the comment. We added explanation of the experimental data. The paper is mostly about the theoretical model and we have clarified this in the introduction.

***Comment #6:***

*In line 328, the authors say that their model predicts linear growth in poor nutrient medium. Is it only approximately linear, as in being transitionary between exponentially growing and stationary states, or can this linearity be demonstrated analytically for some particular nutrient concentration? The authors should elaborate on this*

**Response:** We thank the reviewer for the summary and comment. In the poor nutrient case, we consider growth with protein degradation and ribosome disassembly. In this case, the growth trajectory is not absolutely linear, but can be well approximated as linear in the first hundred cell cycles after the 10^th^ cell cycle (~20 h). To illustrate this point, we performed a long-term cell growth simulation in 2000 h without division (which gives the same growth rate as division case), as is shown in the figure below:

Theoretically, this is not absolutely linear due to degradation, there will be a steady state volume after around 5*10^5^ h. However, this is far out of the realistic time range we are considering. Therefore, in realistic time regime, we can still say the poor-nutrient growth can be well described as linear growth.

To illustrate this point, we have added more discussion in the linear growth case in subsection “Exponential vs linear growth are determined by amino acid import” and SI.

***Comment #7:***

*The effect of t2 on C/N (line 384) comes from the assumption that the ribosome takes the same volume as an individual ribosomal protein. Given that 80 ribosomal proteins go into forming a ribosome, this assumption does not seem correct. This assumption also influences much of the later analysis in the paper.*

**Response:** We thank the reviewer for the comment. In this model, we determine the cell volume by constant osmolarity, which is independent of the volume individual molecules occupied. Most of the cell volume is taken up by water. The cell and nuclear volumes are only dependent on molecule number. This is discussed more extensively by Li. et al, Journal of Cell Sci, 2020 [7]. Therefore, we treat ribosome and protein the same.

***Comment #8:***

***Minor issues:***

*1. In line 14, N/C, or C/N abbreviation should be defined before using it.*

*2. In line 52, it should be Fig. 1(c) and not 1(b). This is also the case in line 98 and line 144.*

*3. Naming the parameter s_1R_ as s_2_, and s_2_ as s_3_ would have made the nomenclature consistent with the numbering for other parameters like n and d, thereby making it easier to remember.*

*4. Fig. 2 should be considerably improved, to show steps like codons in mRNA on which ribosomes move, and amino acid arrival. Otherwise it should be completely removed. In its current form it does not teach much.*

*5. The authors should provide more details on the algorithm/software used to simulate the system of equations.*

*6. In line 321, references 35 and 36 are for enhanced degradation in stem cells. The authors should provide reference for enhanced degradation in yeast and HeLa cells, or discuss why they expect it to be true in these cells as well.*

*7. The authors should mention if same or different values of cytoplasm-nucleoplasm transport coefficients were used in the three cases of Fig.4.*

*8. In line 350, it should be (k)-(o) and not (k)-(l).*

*9. In line 356, it is not clear why increase in t2 should decrease growth rate. The authors should explain it a bit more. I would think that increase in transport of ribosomal protein to nucleus will only enhance ribosome assembly, and can only increase growth rate. Decrease in cytoplasmic ribosomal protein should not reduce the growth rate in their model if the number of mature ribosomes are same.*

*10. The section on protein distribution in individual cells (last section of results) is beyond the central message of this paper and removing that section will improve the sharpness of the central message of the paper.*

*11. Reference/s should be added after the sentence in line 582. Although I feel that this whole paragraph is not necessary for the storyline of the paper.*

**Response:** We thank the reviewer for pointing out the issues. We agreed and have revised the manuscript following the reviewer’s suggestion.

1. We have defined the C/N ratio before line 14.

2. Agreed and done.

3. Agreed and done.

4. We have removed Fig. 2.

5. The MATLAB code is provided on Github.

6. The quiescent state is due to both enhanced degradation and decreased protein synthesis. The balance of these two factors induces quiescence. We have added references for yeast cells [8] and more papers on other mammalian cells [9] to support this statement. For mammalian cell, we just use HeLa cell as an example. Since for many other human cells (e.g., fibroblast cell, stem cell), decreased protein synthesis and enhanced protein degradation are common features in quiescence, we assume this feature could also be generalized to the quiescence of HeLa cells.

7. Values of cytoplasm-nucleoplasm transport coefficients in these three cases are the same. We have made this clearer in the manuscript.

8. Agreed and done.

9. In normal cases, t_2_ increases the growth rate. However, when t_2_ is large, the number of cytoplasmic ribosomal protein is decreased, which decreases cytoplasmic volume. In the meantime, although more ribosomal proteins are transported into nucleus, the ribosome assembly process decreases the total number of molecules in nucleus (80 ribosomal proteins turn into 1 ribosome), and therefore doesn’t increase the nuclear volume. Overall, t_2_ causes decrease in cell volume, thus the growth rate. To make this point clear, we have expanded more on this result in subsection “Effects of protein transport and synthesis on cell growth rate”.

10. The protein distribution part can be applied to the analysis of gene regulatory interaction between different proteins. From single cell proteomics data, our model can be used to infer gene regulatory interactions. Moreover, the protein distribution is directly obtainable from flow cytometry experiments. Therefore, we think it’s a proper extension of our growth model.

11. We have added the relevant references.

**References**

1. Belle, A., Tanay, A., Bitincka, L., Shamir, R. and O’Shea, E.K., 2006. Quantification of protein half-lives in the budding yeast proteome. *Proc. Natl. Acad. Sci. U.S.A.*, 103(35): 13004-13009.
2. Earnest, T.M., Watanabe, R., Stone, J.E., Mahamid, J., Baumeister, W., Villa, E. and Luthey-Schulten, Z., 2017. Challenges of integrating stochastic dynamics and cryo-electron tomograms in whole-cell simulations. *J. Phys. Chem. B*, 121(15): 3871-3881.
3. Gillooly, J.F., Hein, A. and Damiani, R., 2015. Nuclear DNA content varies with cell size across human cell types. *Cold Spring Harb. Perspect. Biol.*, 7(7): a019091.
4. Finka, A. and Goloubinoff, P., 2013. Proteomic data from human cell cultures refine mechanisms of chaperone-mediated protein homeostasis. *Cell Stress and Chaperones*, 18(5): 591-605.
5. Kim, D.H., Cho, S. and Wirtz, D., 2014. Tight coupling between nucleus and cell migration through the perinuclear actin cap. *J. Cell Sci.*, *127*(11): 2528-2541.
6. Van Steensel, B. and Belmont, A.S., 2017. Lamina-associated domains: links with chromosome architecture, heterochromatin, and gene repression. *Cell*, 169(5): 780-791.
7. Li, Y., Konstantopoulos, K., Zhao, R., Mori, Y. and Sun, S.X., 2020. The importance of water and hydraulic pressure in cell dynamics. *J. Cell Sci.*, 133(20), jcs240341.
8. De Virgilio, C., 2012. The essence of yeast quiescence. *FEMS Microbiol. Rev.*, 36(2): 306-339.
9. Zhang, T., Wolfe, C., Pierle, A., Welle, K.A., Hryhorenko, J.R. and Ghaemmaghami, S., 2017. Proteome-wide modulation of degradation dynamics in response to growth arrest. *Proc. Natl. Acad. Sci. U.S.A.*, *114*(48): E10329-E10338.
