# Supplementary material for: The correlation between cell and nucleus size is explained by an eukaryotic cell growth model
Source: PLoS Comput Biol. 2022 Feb 18;18(2):e1009400. doi: 10.1371/journal.pcbi.1009400 (PMC8893647; doi:10.1371/journal.pcbi.1009400)
Supplement: Attachment — Submitted filename: Response letter_PCB_v4.docx [file pcbi.1009400.s005.docx]

**Response to Reviewer #1:**

*In the revised version of "The Correlation Between Cell and Nucleus Size is Explained by an Eukaryotic Cell Growth Model", most of the concerns from reviewers were appropriately addressed. Current, I think it is a nice scientific story that aims at solving fundamental questions in cell biology by computational methods with clear logic. However, I still have some concerns about the stability of the C/N ratio.

In the reply for comment 1, the author states that "we can express growth rate as _ = _ = ", which would already lead to a proportional C/N volume, as a result of the exponential growth with the same exponents. From the current concentration equations the authors listed in the reply, can the Jacobian be calculated to prove the stability of the fixed point solution?

In the line "689", there is an "?" that should not be there.*

**Response:** We thank the reviewer for the comment. The concentration equations as suggested by the reviewer are not closed because we can’t express the volume, C/N ratio and $g_{C,N}$ only in terms of concentration. Therefore, we cannot analyze the C/N ratio stability from a Jacobian just based on concentration equations (We denote C/N ratio as $q$).

$$\left\{ \begin{aligned} \frac{d[A_{C}]}{dt}=\frac{t_{1}\left[ P_{C} \right]}{1+\frac{1}{q}}-(s_{1}+s_{2})\left[ A_{C} \right]\left[ R_{C} \right]-\left[ A_{C} \right]g_{C} \\ \frac{d\left[ P_{C} \right]}{dt}=\frac{s_{1}}{n_{1}}\left[ A_{C} \right]\left[ R_{C} \right]-\left( t_{4}\left[ P_{C} \right]-t_{5}\left[ P_{N} \right] \right)\left[ P_{C} \right]-\left[ P_{C} \right]g_{C} \\ \frac{d\left[ R_{C} \right]}{dt}=t_{3}\left[ R_{N} \right]\left[ P_{C} \right]-\left[ R_{C} \right]g_{C} \\ \frac{d\left[ RP_{C} \right]}{dt}=\frac{s_{2}}{n_{2}}\left[ A_{C} \right]\left[ R_{C} \right]-t_{2}\left[ RP_{C} \right]\left[ P_{C} \right]-\left[ RP_{C} \right]g_{C} \\ \frac{d\left[ RP_{N} \right]}{dt}=t_{2}\left[ RP_{C} \right]\left[ P_{C} \right]q-s_{3}\left[ RP_{N} \right]-\left[ RP_{N} \right]g_{N} \\ \frac{d\left[ R_{N} \right]}{dt}=\frac{s_{3}}{n_{3}}\left[ RP_{N} \right]-t_{3}\left[ R_{N} \right]\left[ P_{C} \right]q-\left[ R_{N} \right]g_{N} \\ \frac{d\left[ P_{N} \right]}{dt}=\left( t_{4}\left[ P_{C} \right]-t_{5}\left[ P_{N} \right] \right)\left[ P_{C} \right]q-\left[ P_{N} \right]g_{N} \end{aligned} \right.$$

Although it’s hard to analytically prove the stability of the C/N ratio, we can test the stability of C/N ratio numerically with different initial conditions and parameters (coefficients of transport, synthesis and degradation). The figure below (Fig. 1) shows C/N ratio curves in both yeast (a) and mammalian (b) cell case with initial conditions varying randomly within 20% of the realistic values. The result numerically shows that the C/N ratio is stable with realistic parameters.

Fig 1. (a) Yeast cell C/N ratio curve with different initial conditions (varying within 20% of realistic values). (b) Mammalian cell C/N ratio curve with different initial conditions.

We also test the stability of yeast cell C/N ratio with different model parameters (varying randomly within 50% of realistic values). In each set of parameters, the initial conditions vary randomly within 20% of realistic values. The figure below (Fig. 2) shows the C/N ratio curves with different parameters:

Fig 2. Yeast cell C/N ratio curve with different parameters and initial conditions. Model parameters are different in (a), (b) and (c), varying randomly within 50% of realistic values.

Therefore, numerical results seem to show that the C/N ratio is stable under different sets of parameters varying within 50% of realistic values.

Similarly, we can test the stability of concentration (here we take cytoplasmic protein as an example) and volume growth rate (Fig. 3). In figure 3 (b), the growth rate is calculated as: $\lambda=\frac{1}{V}\frac{dV}{dt}$, where $V$ is cell volume. To clarify this point, we have added some discussion in part 4 in SI.

Figure 3 (a) Concentration of cytoplasmic protein. (b) Cell volume growth rate. Both quantities are stable when varying initial conditions within 20% of realistic values. In these results, we are using yeast cell parameters.

The reason why the model is generally stable is because the model equations, though nonlinear, are pretty close to linear for the exponential regime. Therefore the solutions are therefore simple exponentials and independent of initial conditions.

**Response to Reviewer #3:**

*The authors have satisfactorily responded to most of my concerns and suggestions. The manuscript reads better now and is more informative. However, we seem to disagree on how to model cellular volume. The authors seem convinced that the cell is a dilute media whose volume is solely determined by its osmolarity, i.e., the cellular volume is proportional to the number of molecules of proteins and ribosomes and is independent of their sizes. This assumption contradicts the modern lore of the cell being a crowded environment and the fact that ribosomes and DNA are significantly large molecules. I should mention that I am mainly aware of the literature on Prokaryotes. I do not know if the authors' assumptions are indeed accurate for Eukaryotes. Also, as there is no experimental evidence of the predicted dependence of cell/nucleus volume on the various parameters studied, it is difficult to assess the correctness of the model. However, I will still recommend acceptance. As the saying goes - all models are wrong, but some are useful. Hopefully, this work will encourage further research on this topic. It will be interesting to see if different modelling approaches predict different outcomes and if experimental observations can differentiate between them.*

**Response:** We thank the reviewer for the comment.
